# Fault Root Cause Tracking of the Mechanical Components of CNC Lathes Based on Information Transmission

**DOI:** 10.3390/s23094418

**Published:** 2023-04-30

**Authors:** Yingzhi Zhang, Guiming Guo, Jialin Liu

**Affiliations:** 1 Key Laboratory of Reliability of CNC Equipment, Ministry of Education, No. 5988 Renmin Street, Nanguan, Changchun 130022, China; 2 School of Mechanical and Aerospace Engineering, Jilin University, No. 5988 Renmin Street, Nanguan, Changchun 130022, China

**Keywords:** fault root cause tracking, signal acquisition, information entropy, net transfer entropy, moving window method, CNC lathe tool

## Abstract

This study proposes a new method for the immediate fault warning and fault root tracing of CNC lathes. Here, the information acquisition scheme was formulated based on the analysis of the coupling relationship between the mechanical parts of CNC lathes. Once the collected status signals were de-noised and coarse-grained, transfer entropy theory was introduced to calculate the net entropy of information transfer between the mechanical parts, after which the information transfer model was constructed. The sliding window method was used to determine the probability threshold interval of the net information transfer entropy between the lathe mechanical parts under different processing modes. Therefore, the transition critical point was determined according to the information entropy, and the fault development process was clarified. By analyzing the information transfer changes between the parts, fault early warning and fault root tracking on the CNC lathe were realized. The proposed method realizes the digitalization and intelligentization of fault diagnosis and has the advantages of timely and efficient diagnosis. Finally, the effectiveness of the proposed method is verified by a numerical control lathe tool processing experiment.

## 1. Introduction

Automation control technology has been widely used with the development of society and the continuous improvement of the technical level in the industrial field. In particular, the mature development of electromechanical-hydraulic integration technology has greatly promoted the realization of intelligent industrial manufacturing. As important carriers of intelligent manufacturing, the healthy and reliable operation of CNC machine tools is crucial to the improvement of industrial production quality and efficiency.

The CNC machine tool has a complex structure, including both CNC and electrical systems as well as mechanical systems. Subsystems of different modules work together to complete various processing tasks. Due to the functional correlation and structural coupling relationship between the subsystems, when a component fails, its abnormal state will be propagated through the correlation relationship between the subsystems, thereby causing cascading faults that ultimately lead to unpredictable economic losses for manufacturing enterprises [1]. For CNC machine tools, the electrical system often has its own alarm system with error instructions, such as an insufficient lubricating oil alarm, a servo motor overheating automatic alarm, and so on. As for the mechanical components that cooperate directly through mechanical coupling (e.g., tool holders and guide rails), most of them do not have a condition monitoring system, making it more difficult to diagnose faults for such mechanical components. Therefore, it is important to conduct research on the fault root cause tracking of the mechanical parts to ensure the safe and orderly progress of the activities involved in production and processing.

Originating from fault diagnostic methods, root tracking technology mainly analyzes the relationship between changes in different component conditions and the propagation and development of root events or causes from the perspective of the system. It also tracks the root causes of faults based on abnormal system conditions. At present, symbolic diagrams [2,3,4], fault trees [5], Petri nets [6,7], and topology models are commonly used to describe the fault propagation process [8,9,10] and rule inference based on experts and prior information [11,12] or on data-driven methods to calculate and discriminate causality according to the process of variables to realize fault root cause identification and location [13,14,15]. Signal-based fault diagnosis methods use measured signals to extract features and make diagnosis decisions based on symptom analysis and prior knowledge [16].

The derivation of rules according to knowledge bases is an important topic in the field of fault root cause tracking. However, these rules come from expert knowledge and require a great deal of experience and knowledge. As modern systems become more complex, the number of process variables increases, occasionally making it difficult to obtain perfect prior knowledge. Concurrently, this kind of method belongs to qualitative reasoning, but lacking quantitative information, it is difficult to judge the strength of the causal relationship between variables, and it is often unable to reveal the deep law of the fault. It is also difficult to choose when there are multiple inference results and when the problem of rule explosion cannot be avoided.

Fault data-based algorithms make certain simplifications or assumptions about the system, leading to deviations or even errors in the results of the algorithm that, in turn, affect the efficiency and accuracy of causality modeling. The fault data used in this method is a record of historical faults. Moreover, the mathematical model of the data-based system depends on the diagnostic system’s structure, and some faults can cause the system structure to change. Therefore, when an abnormal situation occurs, it is difficult to establish a corresponding mathematical model due to the lack of field data.

Signal-based fault diagnosis method. A signal indicates status information obtained by sensors or other devices. With the development of modern computer and measurement technology, it has now become easier to collect, measure, and store process data, enabling people to generate alarms through software and create alarm data by directly setting alarm thresholds for critical variables [17]. Therefore, the causality modeling method using process data and alarm data has been widely studied and developed [18]. For example, for the specific components of CNC machine tools, sensor, signal processing and analysis, and multi-sensor information fusion technologies are used to realize state judgment and fault prediction through certain monitoring and diagnosis models [19,20]. Chen et al. [21] took the original vibration signal of the bearing as input and then used two convolutional neural networks with different kernel sizes to automatically extract different frequency signal features from the original data. They then combined this with long- and short-term memories to identify fault types.

Based on the bearing fault analysis, Gunerkar et al. [22] applied the wavelet transform to process fault signals, after which they established a bearing fault mode diagnosis system combined with the artificial neural network. Hou et al. [23] combined the digraph with the transfer entropy to realize fault detection of an air separation device in the workshop and evaluate fault severity. Al-jonid Khalid et al. [24] fused neural networks with Bayesian networks and then combined particle filter algorithms for real-time condition monitoring and fault diagnosis of CNC lathes. Using the process data provided by PLC, Colasante et al. [25] proposed a fault analysis method based on expert knowledge and a fuzzy logic algorithm. Serin et al. [26] used sensors to collect a large amount of tool vibration, power, stability, and other information using an intelligent algorithm to achieve the detection of tool breakage, vibration, and other states. However, the abovementioned methods are mostly based on a single component. Furthermore, the influence of the fault coupling relationship between the components on the monitoring results is not considered, or the established models are mostly obtained based on subjective reasoning, making it difficult to apply the diagnostic results to actual production.

In the fields of industrial production and biomedicine, many experts and scholars have proposed the method of tracing the root of faults according to the transmission relationship of information. Brian et al. [27] compared transfer entropy and Granger causality and then developed a decision flow to aid users in deciding when to use either. Gang Li et al. [28] proposed a dynamic time-warping-based causality index to dig into the causality relations among candidates and demonstrated this method’s suitability for non-stationary faulty processes. Rui He et al. [29] also proposed an attention-based long-short-term memory (ALSTM) method. In particular, the ALSTM-GC can effectively identify the causality of process variables from varying long-term delays without any prior information. Xie Ping et al. [30] used the variational modal decomposition-transfer entropy method to quantitatively characterize the nonlinear synchronization characteristics and functional associations between cerebral cortex and muscle based on different time frequencies.

The above studies are based on changes in different information data in the production or physiological process, and there is a high correlation between different modules, such as chemical reaction and physiological reaction. For mechanical systems, these connections are weak. Mechanical systems are primarily forces interacting with one another, so we need to find a way to establish a causal relationship between these interactions.

In other words, traditional CNC machine tool fault root cause tracking research mainly relies on experience and prior information in constructing a causal relationship model, combined with rule-based reasoning or the process of calculating variables to determine causality. However, there is a large deviation in the analysis of the fault mechanism and the identification of the fault root cause due to the limitations of experience, system dynamics, and the simplification or assumptions of the algorithm on the system.

As automated machine tools with a relatively simple operation and high machining accuracy, CNC lathes are widely used in the production and processing processes of various fields. Therefore, the current paper analyzes the CNC lathe as the research object. As a multiprocess machining system, the CNC lathe can transmit information such as materials, energy, and control. Similarly, fault propagation can be described as the transfer of information. On this basis, if the information transmission model of the CNC lathe system is identified, the propagation trend and root cause of the fault can be clarified when the physical structure of the system is unknown. Therefore, in the field of engineering practice, research on the fault root cause of CNC lathes based on operating information has important theoretical significance and practical value for the realization of intelligent diagnosis of manufacturing equipment.

The main contributions of this paper are as follows:

(1) This paper uses real-time signals to build a dynamic information transmission model for the mechanical components of CNC lathes. In this model, the information transmission intensity and transmission direction of each connecting side are always changing.
(2) According to information entropy, this paper determines the critical point of mechanical component phase transition and realizes the fault warning of a CNC lathe tool by establishing the corresponding relationship between the tool wear amount and the information entropy during the machining process.
(3) This work proposes a method for determining the probability threshold interval of the net transfer entropy of information by combining the information transmission model. Through comparative analysis, the inflow and outflow of abnormal information are judged, and the root cause of the mechanical component fault of the CNC lathe is traced.

The remainder of this paper is organized as follows: Section 2 elaborates on the fault root cause tracking method for the mechanical components of CNC lathes based on the information transmission model. An application example is illustrated in Section 3. In Section 4, the effectiveness of the proposed method is verified through specific experiments. Finally, the conclusions of this work are presented in Section 5.

## 2. Fault Root Cause Tracking of Mechanical Components of CNC Lathes Based on the Information Transmission Model

This paper takes a certain type of CNC lathe as the research object and proposes a method of fault root cause tracking based on the condition signals of the CNC lathe’s mechanical components. Following the working principle of the CNC lathe, combined with the function-structure mapping relationship, the whole lathe is divided into subsystems, and the coupling relationship of the critical mechanical components in the working process of the CNC lathe is analyzed. Furthermore, a platform for collecting the characteristic information of the mechanical components is built to complete the collection of the condition signals. After denoising and coarse-graining the collected signals, the transfer entropy theory is applied to calculate the net transfer entropy of the information between the mechanical components. Therefore, the net transfer entropy of information is used as an indicator to construct the information transmission model. In addition, the probability threshold interval of information net transfer entropy between mechanically coupled components under different processing conditions is also calculated.

**Figure 1:**
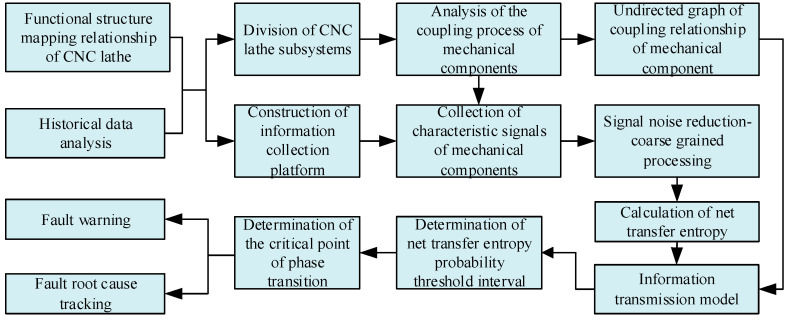
Flowchart of the fault root cause tracking for the mechanical components of the CNC lathe based on the information transmission model.

Furthermore, the fault warning of mechanical components is brought about by combining information entropy to determine the critical point of phase transition. On the basis of the information transmission model of mechanical components, the fault root cause tracking of the mechanical components of the CNC lathe is carried out, the fault root cause is clarified, and the effectiveness of the proposed method is verified through experiments. The findings of this paper are of great significance for the improvement of product quality and processing efficiency during production and processing. The fault root cause tracking flowchart of mechanical components of CNC lathes based on the information transmission model is shown in Figure 1.

### 2.1. Division of the CNC Lathe Subsystem and Analysis of the Coupling Relationship of Mechanical Components

According to the function-structure mapping relationship and working principle of the CNC lathe, this paper divides the subsystem of the lathe and clarifies its main mechanical components. The mechanical coupling process between the critical mechanical components of the CNC lathe is analyzed along with the turning process. Using graph theory knowledge, each mechanical component of the CNC lathe is replaced with a node to form the node set $V=\left\{v_{1},v_{2},\cdots ,v_{n}\right\}$ of the mechanical component. Due to the existence of the coupling relationship between the components, a connecting edge can be used to represent the coupling relationship between the components, from which a set $E=\left\{e_{1\to 2},e_{1\to 3},\cdots ,e_{i\to j}\right\}$ of edges can be obtained. The nodes and edges are combined to form an undirected graph model that can describe the mechanical coupling relationship of CNC lathes: $D_{F}=\left(V,E\right)$, in which *V* and *E* represent the mechanical parts and the direct connection of these mechanical parts, respectively.

### 2.2. Characteristic Signal Acquisition and Processing of the Mechanical Components of the CNC Lathe

Based on the analysis of the coupling relationship between the mechanical components of the CNC lathe, an experimental platform for collecting the mechanical components’ characteristic signals is built. We select appropriate monitoring signals and monitoring points to collect the on-site processing information of the CNC lathe, after which we perform noise reduction and coarse-graining processing on the time series of the information, thus laying the foundation for the construction of the mechanical component information transmission model.

#### 2.2.1. Collection of Characteristic Signals of CNC Lathe

(1) Construction of the characteristic signal acquisition experiment platform

**Figure 2:**
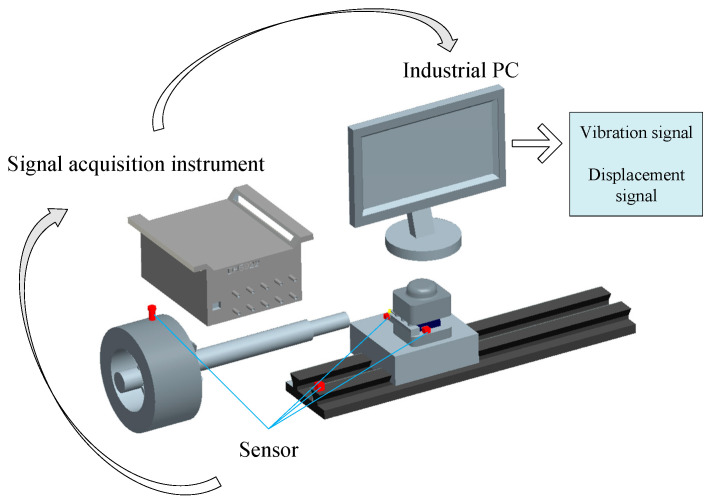
Frame diagram of the signal acquisition experimental platform.

In accordance with the motion characteristics of the different component units of the CNC lathe, signals that can reflect the characteristics of their motion states are selected for monitoring. We built the frame diagram of the signal acquisition experimental platform shown in Figure 2, which mainly includes the CNC lathe, sensors, signal acquisition instruments, signal transmission lines, signal testing, and analysis software.

(2) Type selection of characteristic signals

The purpose of the experiment is to collect characteristic signals that can reflect the movement characteristics of mechanical components during processing. For rotating parts, the rotation accuracy error is used to measure whether they are working normally. Given that the information contained in the radial displacement condition signal can better reflect the working status of the rotating part, most rotating parts use non-contact displacement sensors to monitor radial runout in real time. However, during processing, the non-rotating parts often use vibration sensors to collect vibration signals as information indicators that reflect their working status.

(3) Selection of characteristic signal monitoring points

Considering the complexity of the structure of the CNC lathe, to avoid the problem of a signal unable to accurately reflect the characteristics of a component’s movement state due to the unreasonable selection of the monitoring point during the signal acquisition, this paper must select the appropriate monitoring point before the signal acquisition. In the current paper, the EMD decomposition algorithm [31] and Fourier transform [32] are used to process the collected signals. Furthermore, the best monitoring point is determined based on the signal characteristics of each monitoring point after processing and the strength of the signal’s ability to provide feedback information.

#### 2.2.2. Signal Denoising Processing Based on Wavelet Transform

**Figure 3:**
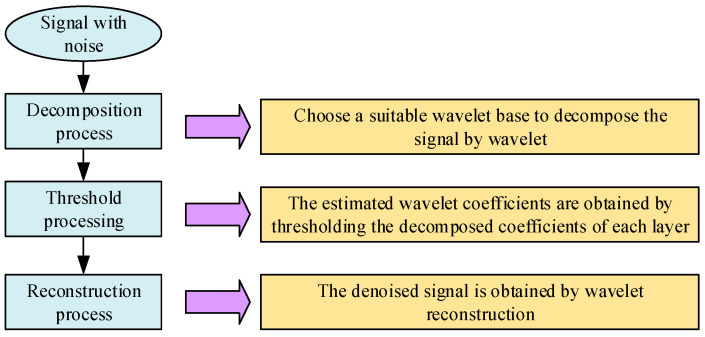
Wavelet threshold denoising flowchart.

The signal collected by the sensor must be processed for noise reduction in order to effectively suppress the interference of irrelevant variables on the original data, so that the time series signal can truly reflect the dynamic characteristics of the component unit. The wavelet de-noising algorithm [33] is a widely used signal de-noising method. Thus, the present paper uses a wavelet threshold de-noising algorithm to de-noise the collected condition signals of the lathe’s mechanical components. The de-noising process is shown in Figure 3.

The wavelet threshold denoising algorithm [34,35] is based on the variable scale property expansion of the discrete wavelet transform. When a signal is subjected to the discrete wavelet transform, the energy of the original signal is concentrated in the wavelet coefficients. By setting the threshold, the wavelet coefficients that meet the threshold requirements are reconstructed, from which the original signal after denoising is obtained. The noise signals that cannot be concentrated are scattered in the wavelet domain. These are eliminated if they cannot meet the preset threshold requirement, thereby realizing the separation of the noise signal.

The process of continuous wavelet transform is shown in Equation (1):

(1) $$W_{f}(a,b)=\int_{-\infty }^{\infty }f(t)\bar{\psi_{a,b}(t)}dt$$

where $\psi_{a,b}(t)$ is the basis function of the wavelet transform, $\bar{\psi_{a,b}(t)}$ is the conjugate of $\psi_{a,b}(t)$, and f(t) is the original signal.

(2) $$\psi_{a,b}(t)=\frac{1}{\sqrt{\left|a\right|}}\psi (\frac{t-b}{a})$$

In Equation (2), a is a scaling parameter, b is a translational scaling parameter; a,b∈R;and a≠0.

The output signal directly sampled by the sensor is actually a discrete signal, so it is necessary to discretize the continuous wavelet transform. Here, we take $a=2^{m};\;b=2^{m}n$, *m*; and n as natural numbers. Therefore, the discrete wavelet transform process can be expressed as:

(3) $${W_{f}}^{\prime }(m,n)=\frac{1}{\sqrt{\left|2^{m}\right|}}\int_{-\infty }^{\infty }f(t)\bar{\psi \left(\frac{t-2^{m}n}{2^{m}}\right)}dt$$

#### 2.2.3. Feature Signal Coarse-Grained Processing Based on PAA Algorithm

The signal data collected during lathe processing covers a wide range of information and can contain all the information reflecting the component’s movement state. However, the high-dimensional characteristics of time series are not conducive to data feature extraction and analysis. Therefore, the time series signals collected under the machining state of the lathe must undergo coarse-grained processing to reduce data complexity.

The piecewise aggregate approximation (PAA) algorithm is a commonly used algorithm for processing time series. Unlike the common wavelet transform, Fourier transform, and other processing methods, it does not change the original performance characteristics of the sequence. The time series after PAA processing remains a time domain diagram, in which time is the abscissa.

The main features of the PAA algorithm are as follows:

(1) It can reduce the dimensionality of the time-series signal collected by the sensor.
(2) The time series can retain the characteristics of the original data after coarse-grained processing.
(3) It has good inclusiveness for noisy data and a certain denoising ability.
(4) The coarse-grained time series is closely related to the trend of the original time series.

Through the use of the PAA to reduce the dimensionality of the time series, the signal $X\left(t\right)$ is divided into equal-length sub-sequences ${\bar{x}}_{i}$, and ${\bar{x}}_{i}$ is replaced by the mean value of the sequence. As such, the time series $\bar{X}\left(t\right)$ after dimensionality reduction can be obtained according to Equation (4):

(4) $${\bar{x}}_{i}=\frac{1}{w}\sum_{j=w\times \left(i-1\right)+1}^{w\times i}x_{i}$$

where $x_{i}$ is the original time series, and w is the width of the sub-sequence.

The length of w in the PAA dimensionality reduction process determines the degree of the signal’s coarse-graining after the dimensionality reduction. By denoising and coarse-graining the time series signal, a low-dimensional time series that is easier to calculate can be obtained, and the new time series can retain the characteristic information of the original time series.

### 2.3. Construction of the Information Transmission Model of the Mechanical Components of the CNC Lathe Based on Transfer Entropy

#### 2.3.1. Related Theories of Transfer Entropy

The concept of “entropy” originated from the description of the parameters of the state of material in thermodynamics. Since Shannon first proposed information theory in 1948, it has become the focus of numerous studies [36]. As a powerful tool to quantify information, information entropy is widely used in various fields, such as cybernetics [37], probability theory [38], and life sciences [39]. Although information entropy can describe the state of a single variable well, it is difficult to measure the information transfer relationship between multiple variables. Therefore, Schreiber combined information entropy with time-delay interactive information to propose the concept of transfer entropy [40]. Transfer entropy can be used to describe the causal relationship between multiple variables.

Information entropy refers to the amount of information that measures the real-time working status of each mechanical component of a lathe during the working process. For example, the vibration signal collected by the sensor is essentially a time series with some randomness and uncertainty. From the definition of information entropy, we can see that for each segment of a vibration signal, the magnitude of its amplitude is regarded as a measure of uncertainty that changes with the time series. The probability that the amplitude $x_{i}$ corresponding to a different time node i appears in the entire time series can be measured by $P\left(x_{i}\right)$, and the information amount of $x_{i}$ corresponding to the node i at any time can be expressed by the following equation:

(5) $$\ln\left(x_{i}\right)=-\log_{2}P\left(x_{i}\right)$$

where $0\leq P\left(x_{i}\right)\leq 1$, $\sum_{i=1}^{n}P\left(x_{i}\right)=1$.

For the entire time series, the mathematical expectation of the variable amplitude $x_{i}$ can be used to describe the information entropy of the time series. Thus, the information entropy $H\left(X\right)$ of the entire vibration signal X can be expressed as follows:

(6) $$H\left(X\right)=E\left[\ln\left(x_{i}\right)\right]=-\sum_{i=1}^{n}p\left(x_{i}\right)\log_{2}p\left(x_{i}\right)$$

Information entropy, as a measure based on the probability of mathematical statistics, also has its own unique mathematical properties [41], as described below.

(1) Additivity

For two independent variables, namely, the information entropy $H\left(X\right)$ of X and the information entropy $H\left(Y\right)$ of Y, the relationship between the information entropy $H\left(X,Y\right)$ of its joint variable XY can be expressed as:

(7) $$H\left(X,Y\right)=H\left(X\right)+H\left(Y\right)=-\sum_{i=1}^{n}\sum_{j=1}^{n}P\left(x_{i},y_{j}\right)\log_{2}P\left(x_{i},y_{j}\right)$$

(2) Symmetry

The magnitude of $H\left(X\right)$ has nothing to do with the order of its probability vector $P_{1},P_{2},\cdots ,P_{n}$, namely:

(8) $$H\left(P_{1},P_{2},\cdots ,P_{n-1},P_{n}\right)=H\left(P_{n},P_{n-1},\cdots ,P_{2},P_{1}\right)$$

Entropy can be measured by the mathematical expectation of the amount of information, and the amount of information between two events that have mutual influence can be measured by conditional entropy. In other words, the uncertainty of an event X under the condition of a known event Y can be expressed by conditional entropy $H\left(X\left|Y\right.\right)$, as shown in Equation (9).

(9) $$H\left(X|Y\right)=-\sum_{i=1}^{n}\sum_{j=1}^{n}P\left(x_{i},y_{i}\right)\log_{2}P\left(x_{i}|y_{i}\right)$$

#### 2.3.2. Transfer Entropy Calculation Model

The calculation premise of the transfer entropy is that the time series object must satisfy the Markov process. The Markov process is derived from the Markov chain, and its most important feature is that it has no aftereffect [42]. The characteristic signal in the working process of the CNC lathe is a random, uncertain signal. The characteristic parameter of each time point has nothing to do with the signal characteristic parameter before this time node. Rather, it is an independent random process that satisfies the Markov characteristic of having no aftereffect.

For vibration signals, if the probability of the amplitude at a given time $\left(n+1\right)$ is only related to the previous k time points, this vibration time series can be called a k-order Markov process [43]. The Markov process of a vibration signal X at a given time t can be described as:

(10) $$P\left(x_{i}|x_{i}^{k}\right)=P\left(\left(x_{i}\left(n+1\right)\right)|x_{i}\left(n\right),x_{n-1},\cdots ,x_{i}\left(n-k+1\right)\right)$$

From the definition of information entropy, it can be seen that for two characteristic signals X and Y that satisfy the Markov process, if the transition probability of the influence of signal Y on signal X is P, then the information coupling relationship between processes x and y can be expressed as [44]:

(11) $$T_{y\to x}(x(1)|x^{k},y^{t}(\tau ))=\int p(x(1),x^{\left(k\right)},y^{\left(t\right)}(\tau ))\log_{2}\left\{\frac{p(x(1)x^{\left(k\right)},y^{\left(t\right)}(\tau ))}{p(x(1)|x^{\left(k\right)})}\right\}\times dx(1)dx^{\left(k\right)}dy^{\left(t\right)}$$

where $T_{y\to x}$ is the transfer entropy of process y to x, and k and l are the orders of process x and process y, respectively.

For CNC lathes in an operating state, the transfer entropy can be used to quantify the degree of influence of the state between mechanical components. For example, when the tool holder is in the stroke of the empty tool, the tool holder and the spindle are not in contact. Although the former is actually connected with the latter through other structures of the machine tool body, the influence of the working state of the tool holder on the spindle should be zero from the point of view of the mechanical coupling relationship. At this time, the dynamic process y cannot provide influence information on the future state changes of the dynamic process x, namely:

(12) $$T_{y\to x}=0$$

When the workpiece is being processed, the mechanical coupling relationship between the tool holder and the spindle is produced by cutting the workpiece through the tool. Assuming that a sensor A is arranged at the tool holder and a sensor B is arranged at the spindle. The signal can reflect the current working state of the subsystem. According to the definition of transfer entropy, the expression of transfer entropy between the time series signals at A and B is shown as follows:

When the workpiece is being processed, the mechanical coupling relationship between the tool holder and the spindle is produced by cutting the workpiece through the tool. Assuming that a sensor A is arranged at the tool holder and a sensor B is arranged at the spindle, the signal can reflect the current working state of the subsystem. According to the definition of transfer entropy, the expression of transfer entropy between the time series signals at A and B is expressed as follows:

(13) $$T_{A\to B}=\sum_{A_{i+1},A_{i}^{\left(k\right)},B_{i}^{l}}p(A_{i+1},A_{i}^{\left(k\right)},B_{i}^{\left(l\right)})\log_{2}\frac{p(A_{i+1}|A_{i}^{\left(k\right)},B_{i}^{\left(l\right)})}{p(A_{i+1}|B_{i}^{\left(k\right)})}$$

where $T_{A\to B}$ is the transfer entropy of the process A to B. Under the first-order Markov process, the calculation model of transfer entropy can be simplified as follows:

(14) $$T_{A\to B}=\sum_{A_{i+1},A_{i},B_{i}}p(A_{i+1},A_{i},B_{i})\log_{2}\frac{p(A_{i+1}|A_{i},B_{i})}{p(A_{i+1}|B_{i})}$$

The transfer entropy of A to B is essentially the change of A to the uncertainty of B information. This can also be understood as the degree of influence influence of the change of the time series A on the change of the time series B.

#### 2.3.3. Construction of the Information Transmission Model of the Mechanical Components of the CNC Lathe Based on Transfer Entropy

Given that transfer entropy can reflect the mutual influence relationship between different time series, this paper introduces transfer entropy theory and combines the undirected graph model of the coupling relationship of the mechanical components to construct the information transmission model of the CNC lathe’s mechanical components. The net transfer entropy TE of the information between mechanically coupled components is used as the information transfer intensity of the connecting edge, and the direction of the connecting edge is determined by the positive and negative values of TE. The following focuses on the calculation of the net transfer entropy of information between mechanical components:

According to the calculation Equation (13) of transfer entropy, the main input for calculating transfer entropy includes the following:

(1) Real-time time series signals of two nodes *A* and *B*, and
(2) delay time series of node *B*.

To avoid further loss of information in the process of calculating the transfer entropy of the time series after coarse-grained processing, the time delay τ can be determined as 1, which refers to the time length of a data point after coarse-grained processing [45].

This paper assumes that the collected signal time series all satisfy the first-order Markov process. Using the noise-reduced and coarse-grained time series as inputs, the calculation model of transfer entropy between mechanical coupling components of the CNC lathe is established in MATLAB. The calculation process is as follows:

(1) The time series signals of the collected mechanical components are A(t) and B(t).
(2) As the original signal will be mixed with noise interference, noise interference will affect the intensity of information transmission, but it will not change the direction of information transmission. Therefore, it is necessary to perform noise reduction processing on the time series signal to obtain time series signals $A^{\prime }(t)$ and $B^{\prime }(t)$ with a high signal-to-noise ratio.
(3) Coarse-grained processing of time series. On the premise of maintaining the original characteristics of the time series, the dimension of the time series is reduced to obtain the coarse-grained time series signals $\bar{A^{\prime }}(t)$ and $\bar{B^{\prime }}(t)$.
(4) Calculation of net transfer entropy $TE_{A\to B}$. Here, we define $T_{B\to A}-T_{A\to B}$ as the net transfer entropy [46] and then calculate the net transfer entropy of information between A and B according to Equation (15):
  (15) $$TE_{A\to B}=T_{B\to A}-T_{A\to B}$$
  If $T_{B\to A}-T_{A\to B}>0$, it means that the information flows from B to A, and vice versa (i.e., information flows from A to B).
(5) The information transfer intensity between A and B can be quantitatively described by the size of the net transfer entropy $TE_{A\to B}$ between the two components. The positive or negative aspect of the net transfer entropy can represent the direction of the connection edge.

### 2.4. Fault Root Cause Tracking of the Mechanical Components of the CNC Lathe Based on the Information Transmission Model

#### 2.4.1. Determination of the Probability Threshold Interval of the Net Transfer Entropy of Information between Components under Different Processing Conditions

Given that the movement relationship between the components is constantly changing during processing, the intensity and direction of information transmission are not constant. However, under normal processing conditions, the net transfer entropy of information should fluctuate within a fixed range. When such entropy exceeds a certain threshold interval, it means that the mechanical components corresponding to the connection edge may appear abnormal. Therefore, it is necessary to divide the fluctuation interval of the normal information net transfer entropy of each connecting edge under normal working conditions. By comparing whether the net transfer entropy of the measured information is within the threshold interval, it can be ascertained whether the connection edge has fault propagation.

Meanwhile, CNC lathes are mainly used to process the outer circle part of workpieces. Based on its different working modes, its movement modes can be divided into the following two types:

(1) One-way movement in the Z direction, and
(2) combined movement in the X and Z directions.

The machining process can be divided into the above two cutting modes. In different cutting modes, the relative motion and mutual force relationship between the tool and the workpiece are different. In addition, different interactions can inevitably lead to different types of interactive information. According to the principle of transfer entropy, the amounts of information transferred in processing vary under different processing modes.

Liu et al. [47] and Sun et al. [48] applied the moving window method and cumulative probability density, respectively, to calculate the probability threshold interval of the net transfer entropy of information at different connected edges. However, because the difference in the transfer entropy of different processing states is not considered, the probability threshold interval of the net transfer entropy of information lacks pertinence. Therefore, this paper collects and analyzes the processing signals of CNC lathes under different processing modes and determines the probability threshold interval $\left[TE_{L},TE_{U}\right]$ of the net transfer entropy of the information in the normal state, thus laying the foundation for the realization of fault root cause tracking.

Taking the connecting edge between the guide rail and the tool holder as an example, the moving window method is used to determine the fluctuation interval of the net transfer entropy of information in the two cutting processing modes. The window width of the moving window method can be divided according to a complete processing cycle comprising 300 sampling points. Here, we take twice the minimum processing cycle as the window width and 80% of the window width as the step length [49]. The parameter selection of the moving window is shown in Table 1.

**Figure 4:**
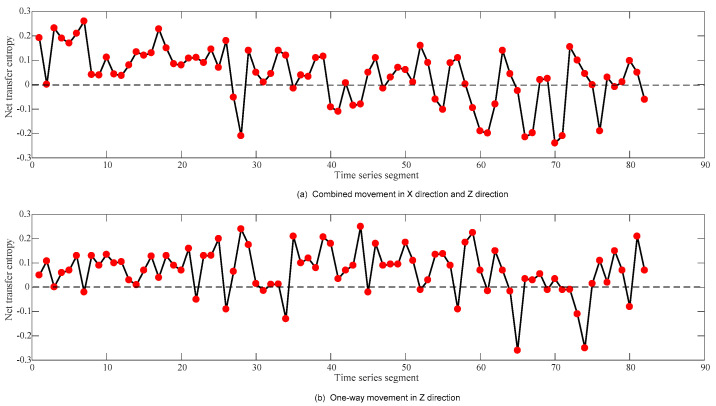
The trend chart of the information net transfer entropy values of the tool holder—guide rail under two processing conditions.

The information net transfer entropy values of the tool holder—guide rail under two different processing conditions are shown in Figure 4.

**Figure 5:**
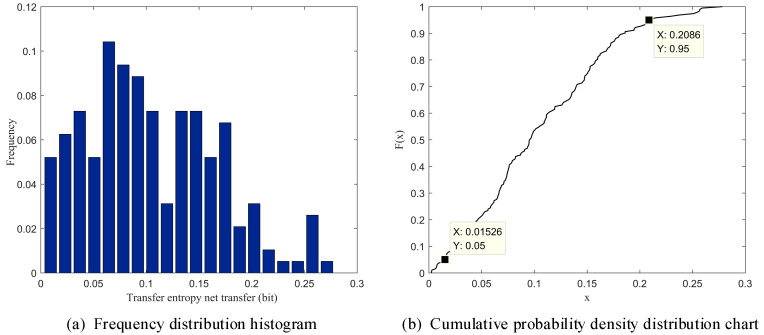
Statistical analysis diagram of the net transfer entropy of information between the tool holder and guide rail.

The frequency distribution histogram and the cumulative probability density distribution diagram of the information net transfer entropy between the tool holder and guide rail in the Z direction are both shown in Figure 5.

A cumulative probability density distribution is often used for processing large numbers of uncertain data for interval distribution statistics. In this paper, we select a lower boundary of 0.05 and an upper boundary of 0.95 as the confidence interval (CI) for the numerical probability density [50]. By combining the cumulative probability density distribution diagram, we can determine the probability threshold interval of the net transfer entropy of the information between the tool holder and guide rail corresponding to the Z-direction motion processing state, which has a value of $\left[0.0153,0.2086\right]$.

#### 2.4.2. Determination of the Critical Point of Phase Transition Based on Information Entropy

**Figure 6:**
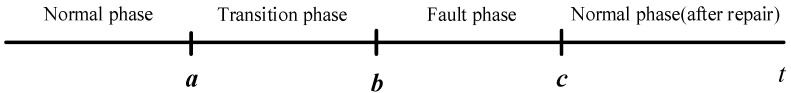
Fault evolution process.

The component has a phase transition process from the normal to the fault phase, as shown in Figure 6. As can be seen, time node *a* is the critical point between the normal phase and the transition phase, while *b* is the point where the fault occurs, that is, the starting point of the component fault. After the maintenance intervention at time point *c*, the original component is restored to its normal working state. The state from time a to time *b* is considered an abnormal state. However, during the period from a to *b*, the components still show a normal working state, but in fact they are already in a transition stage from the normal state to the fault state, which can be defined as the phase transition process of the fault.

Information entropy, as an important feature of the component state, can be used to reflect the change process of the component’s working state. For example, the information transfer relationship in the time period $\left(a,b\right)$ is different from that between the $\left(b,c\right)$, and this characteristic becomes more obvious as the degree of fault increases. Based on this characteristic, this paper combines information entropy to identify the fault phase transition process of mechanical components. This technology can take measures before the components are in the fault phase to suppress the occurrence of faults and provide a reference for component replacement and preventive maintenance.

#### 2.4.3. Fault Warning of the Mechanical Components of CNC Lathes Based on the Critical Point of Phase Transition

**Figure 7:**
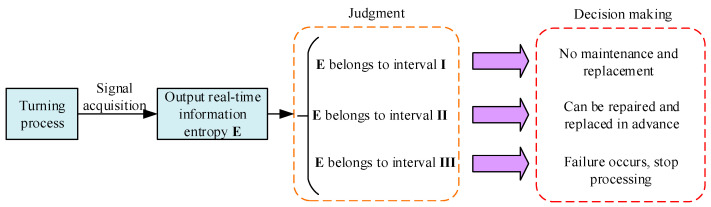
Fault-warning flow chart of mechanical components.

The fault warning of mechanical components is mainly based on the critical point of fault phase transition. This paper collects the characteristic signals of each component for a complete life cycle and divides the information entropy according to the collected signals. These are divided into normal phase I, transition phase II, and fault phase III, after which a fault warning database for this component is established. After that, the information entropy can be calculated by interval sampling during the processing, and the results are compared with the database to realize real-time state monitoring of mechanical components. The specific fault warning process is shown in Figure 7.

The component fault warning can be realized by performing real-time or interval sampling monitoring of the state information entropy of each mechanical component in the processing and by comparing the sampling results with the established fault warning database. Accordingly, repairing or replacing components before they fail can ensure the orderly progress of production, improve the processing quality of workpieces, and prevent the further development and propagation of faults.

#### 2.4.4. Fault Root Cause Tracking of the Mechanical Components of CNC Lathes Based on the Information Transmission Model

**Figure 8:**
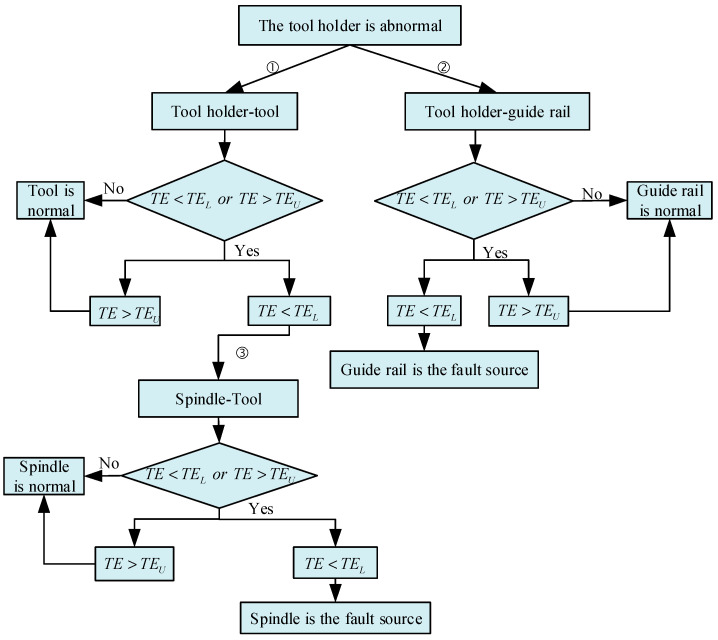
Flow chart of the fault root cause tracking of the tool holder.

The fault phase transition identification of mechanical components can clarify the fault evolution process. Here, the fault can be eliminated before it occurs by tracking the root cause of the fault in the mechanical components in the transition phase. This paper takes the fault root tracking process when the tool holder has abnormal performance as an example, as shown in Figure 8. As can be seen, the tool holder is used as the starting node, and the net transfer entropy of the information between the nodes directly and indirectly related to the tool holder is calculated according to the information transmission model. It performs the calculation one by one along the connection edges where abnormal information is transmitted. It also analyzes layers by layers and judges whether there is abnormal information transmission until the starting node of abnormal information transmission is found, which is the root cause of the fault.

When a component has an abnormal state, we must compare whether the net transfer entropy of the adjacent connection edge exceeds the probability threshold interval following the tracking flowchart shown in Figure 8. This is performed in order to determine whether there is abnormal information flowing in or out. If there is abnormal information flowing in, it means that the current component is affected by the abnormal state of other components and is not the starting point of the fault. It is necessary to further track the root cause of the fault. If only the outflow of abnormal information is found, it means that the current component is the root cause of the fault.

The combination of mechanical component fault warning and fault root cause tracking methods can prevent or reduce the occurrence of mechanical component faults on CNC lathes. This can provide guidance for the formulation of maintenance and replacement strategies for the lathes’ mechanical components during production and processing.

## 3. Application Example

**Figure 9:**
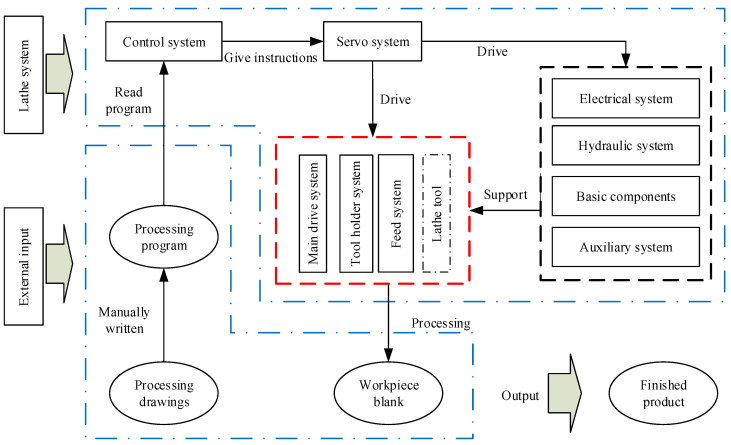
Schematic diagram of the CNC lathe working process.

This experiment uses a certain model of a CNC lathe in good condition as the research object. According to the working principle of the lathe, it is divided into modules to form different subsystems. The results of the division are shown in Table 2. On the basis of the division of each system of the lathe in Table 2, combined with the machining principle of the CNC lathe, the working process of the CNC lathe is decomposed, as shown in Figure 9.

**Figure 10:**
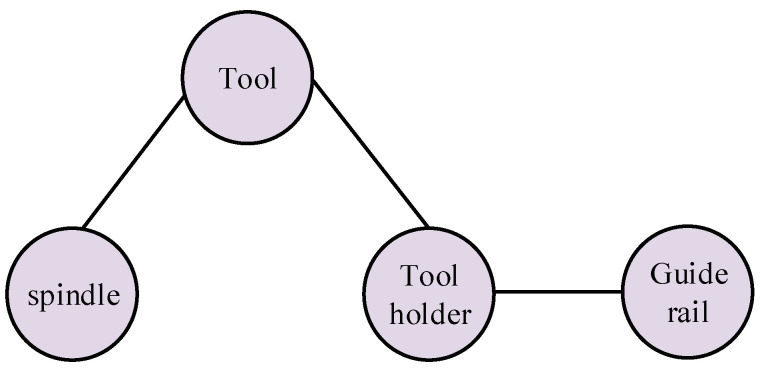
Undirected graph model of the mechanical coupling relationship of a CNC lathe.

It can be seen from Figure 9 that the mechanical modules of the CNC lathe mainly include a spindle system, a tool holder, and a guide rail system. The state of the tool will have a direct impact on the surface quality of the workpiece because such a tool is a component that directly contacts the workpiece for cutting. Therefore, this paper uses the tool as an independent mechanical component to analyze the fault propagation relationship. The four components of the spindle (D), tool (T), tool holder (TS), and guide rail (F) form a mechanical coupling relationship with each other due to the force of the machining process. Combining this information with the knowledge of graph theory, the undirected graph model of the mechanical coupling relationship of the CNC lathe can be obtained, as shown in Figure 10.

Using the frame diagram of the signal acquisition experiment platform shown in Figure 2, we analyze the dynamic information characteristics of each mechanical component and select the appropriate signal type, as shown in Table 3.

**Figure 11:**
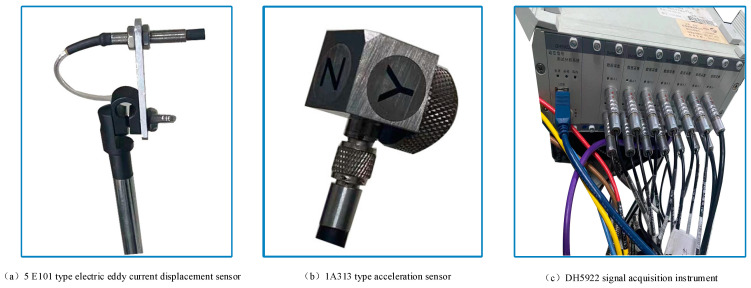
Experimental sensors and signal acquisition equipment.

According to the type of acquisition signal, the 5E101 type electric eddy current displacement sensor (as shown in Figure 11a) is selected for displacement signal acquisition, and the 1A313 type acceleration sensor (as shown in Figure 11b) is chosen for vibration signal acquisition. The specific parameters of each type of sensor are shown in Table 4.

This paper uses the DH5922 signal acquisition instrument (as shown in Figure 11c) and the supporting DHDAS-type signal analysis software platform, combined with the sensor, to realize the real-time monitoring and recording of signals. Furthermore, the analysis software can perform basic processing, such as spectrum analysis, on the signal.

**Figure 12:**
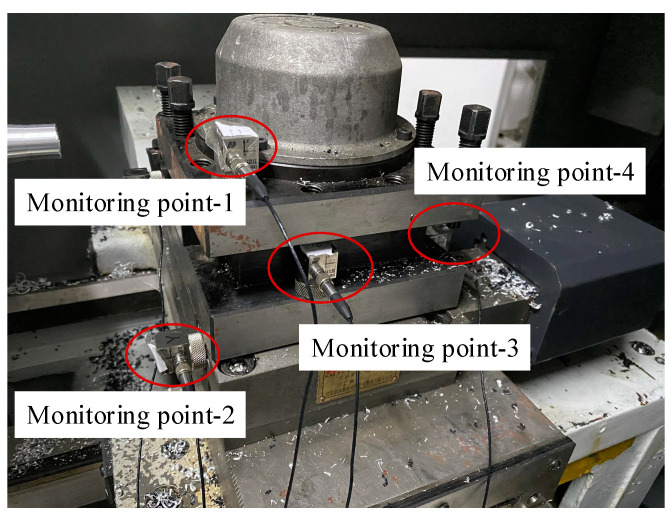
Distribution diagram of the vibration sensor during the selection of tool holder monitoring points.

To obtain a more accurate and complete signal, the best monitoring point must be selected. This paper uses the tool holder as an example. As can be seen, four vibration sensors are arranged in each part of the tool holder, as shown in Figure 12.

The vibration signals generated during the tool change process of the tool holder were collected. The signals collected by the four vibration sensors were analyzed by EMD decomposition and frequency spectrum analysis in order to compare and identify the differences in the vibration signal characteristics of various measuring points. The DHDAS dynamic acquisition and analysis system is used to record the vibration signals of the four different measuring points of the tool holder. The signal characteristics of each measuring point are summarized as shown in Table 5.

**Figure 13:**
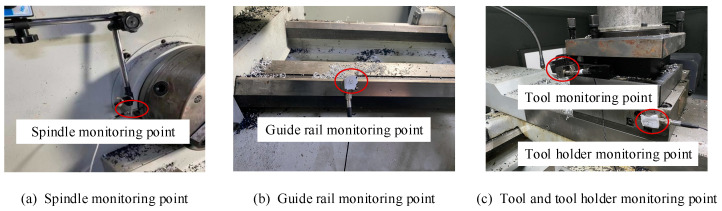
Measurement point distribution diagram of each component.

By comparing the signal characteristics of each part of the tool holder, we can see that the vibration signal frequencies of Points 1, 3, and 4 are concentrated, which only reflects part of the vibration characteristics of the tool holder. Meanwhile, Point 2 covers a wide frequency range, and the root mean square value of its signal is significantly larger than those of the other three measuring points, indicating that it covers comprehensive information and has a stronger ability to provide feedback. To prevent information redundancy from increasing the calculation amount, Point 2 is selected as the measuring point of the tool holder vibration signal, while Points 1, 3, and 4 are discarded. Similarly, after screening the measuring points, the sensor distribution of the spindle, guide rail, tool, and tool holder is shown in Figure 13.

This experiment mainly collects the characteristic signals of each component in the case of turning the outer circle. The specific processing parameter settings are shown in Table 6.

**Figure 14:**
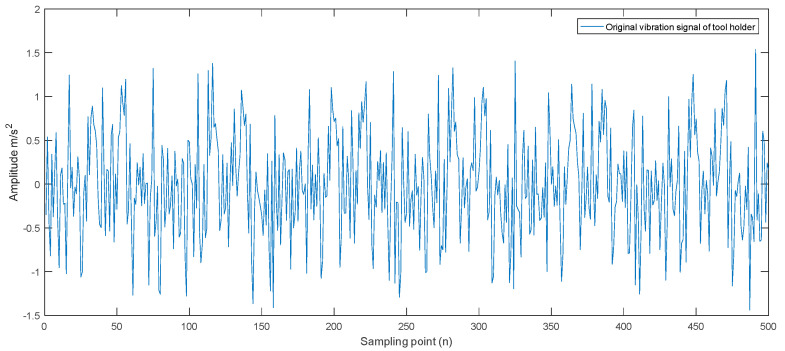
The original vibration signal of the tool holder.

**Figure 15:**
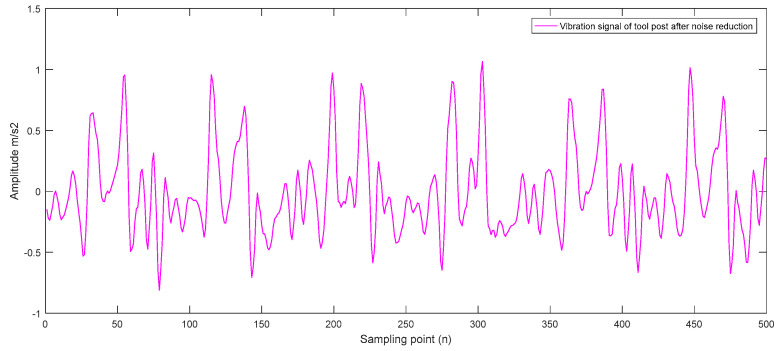
Vibration signal of the tool holder after noise reduction.

Next, we perform wavelet denoising processing on the real-time characteristic signals of the collected mechanical components. This paper selects the db13 wavelet base to reduce the noise of the collected signals based on the vibration characteristics of the CNC lathe machining process. Taking the collected vibration signal of the tool holder as an example, the original vibration signal of the tool holder is shown in Figure 14, and the vibration signal obtained after wavelet noise reduction processing is shown in Figure 15.

According to the characteristics of the vibration signal of the lathe, the time domain width of the upper and lower peaks or troughs bounded by 0 can be found d=dY−dX=0.001 mm, and the signal sampling frequency is 5000 HZ. To keep the characteristic signals of the original sequence $X\left(t\right)$ as much as possible after dimensional reduction, the peak and trough of the wave should be avoided in the same coarse-grained interval. In this paper, the width of w is selected to be 0.001 mm, that is, five sampling points, and PAA is used to reduce the dimensionality of the time series.

**Figure 16:**
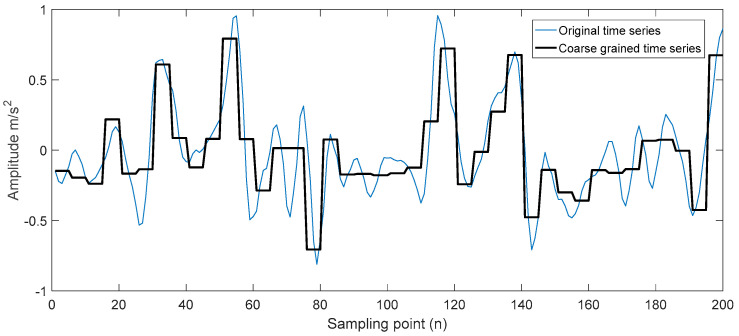
Coarse-grained processing of the tool holder vibration signal.

Furthermore, taking the tool holder as an example, the time series is obtained after the coarse-grained dimensionality reduction processing of the tool holder vibration signal, and the result is shown in Figure 16.

According to the calculation steps of transfer entropy, we calculate $TE_{D\to T}$, $TE_{T\to TS}$, and $TE_{F\to TS}$ using Equations (13)–(15). We collect the time series signals of the spindle, tool, tool holder, guide rail, and other mechanical components during the processing when the time length is *t* and then calculate the net transfer entropy value of the information of each connecting side, as shown in Table 7.

**Figure 17:**
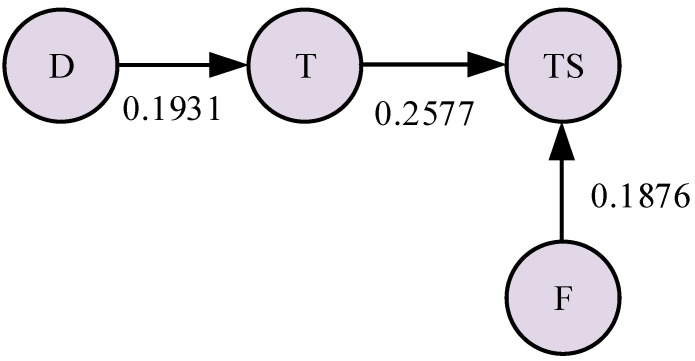
Information transmission model of the mechanical components of the CNC lathe.

The net transfer entropy of information for each connected edge obtained in Table 7 is taken as the information transfer intensity value. Here, we combine $TE_{D\to T}$, $TE_{T\to TS}$, $and\;TE_{F\to TS}$ with the undirected graph of the mechanical coupling relationship of the CNC lathe in Figure 10 to obtain the information transmission model of the mechanical components, as shown in Figure 17.

According to Figure 17 and the information net transfer entropy probability threshold interval, we measure the probability threshold interval of the information net transfer entropy of other connecting edges in two different processing states. The results are shown in Table 8.

From Table 8, we can obtain the probability threshold interval of the information net transfer entropy of the connecting edge in the normal state. When the information net transfer entropy value of the connected edge exceeds the threshold interval, there is a possibility of abnormal signal inflow. Therefore, the determination of the probability threshold interval of the information net transfer entropy can provide guidance for the fault warning and fault root cause tracking of the mechanical components of the CNC lathe.

**Figure 18:**
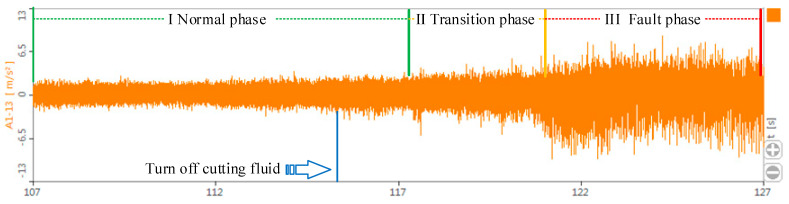
Vibration signal map of the tool fault development process.

In the machining process of the CNC lathes, the tool is a component that directly interacts with the workpiece, and its performance directly affects the quality of the machined parts. Thus, we take the tool processing process as an example to identify the fault phase transition process. In particular, we select the external turning tool model YBC251, collect the vibration signal of the turning tool from normal to fault during the machining process, and obtain the vibration signal map of the tool fault development process, as shown in Figure 18.

**Figure 19:**
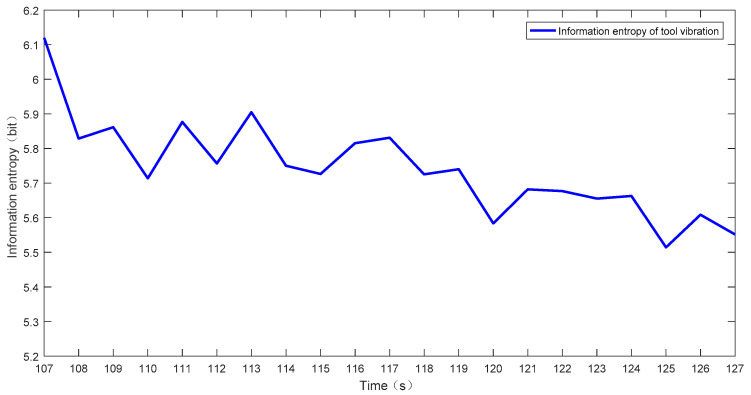
Trend chart of the tool information entropy changes with usage time.

According to Figure 18, we can see that the vibration amplitude of the tool gradually changes with processing time, particularly the very obvious vibration amplitude in the time interval before reaching the fault. By calculating the information entropy of the tool in the machining process, a trend chart of the change of the tool information entropy with use time is drawn, as shown in Figure 19.

As can be seen from the trend chart of tool information entropy that changes with time of use, there are differences in tool information entropy in different states. Furthermore, the trend chart changes significantly from the normal phase to the fault phase. IOS stipulates that the wear band width *VB* measured on the flank surface of the cutting tool with 1/2 of the back cutting depth of the tool is used as the blunt standard [51]. In accordance with the national standard on the fault of general external turning tools, when the tool flank wear band width *VB* = 0.3 mm, the tool can be judged to have a fault [52].

**Figure 20:**
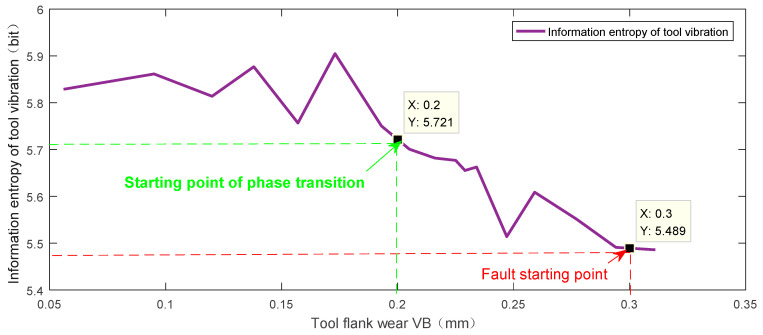
Trend chart of tool information entropy changing with wear amount (VB).

Generally, when the wear amount of the tool is within the range of 0.05–0.1 mm, it is judged as initial wear [53]. This paper divides the wear band width *VB* and the fault process. The wear amount *VB* value is defined as the normal phase in the interval $\left[0,0.2\right]$ mm and the transition phase in the interval $\left[0.2,0.3\right]$ mm. When *VB* exceeds 0.3 mm, it is regarded as entering the fault phase. The trend chart of the tool information entropy changing with the tool wear amount *VB* can be obtained by using multiple experiment interval sampling measurement records and calculating the size of the corresponding information entropy value for different wear band widths. The resulting trend chart is shown in Figure 20.

From Figure 20, we can see the critical point *VB* = 0.2 mm in the normal phase and the critical point *VB* = 0.3 mm in the transition phase, with the corresponding entropy values of E1 = 5.721 and E2 = 5.489, respectively.

In the past, measuring the wear band entailed frequent disassembly and installation of the tool and required the use of a micrometer for measurement. In this paper, by selecting the information entropy value E2 = 5.489 corresponding to *VB* = 0.3 mm as the fault warning threshold, only the tool vibration signal must be collected during the machining process to calculate the information entropy. The tool fault warning can be realized by comparing the results with the threshold.

## 4. Verification of the Fault Root Cause Tracking Method Based on an Accelerated Life Experiment

**Figure 21:**
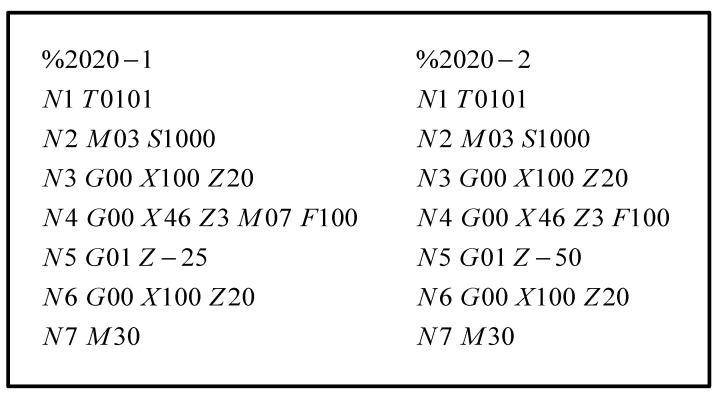
The cutting processing program code.

**Figure 22:**
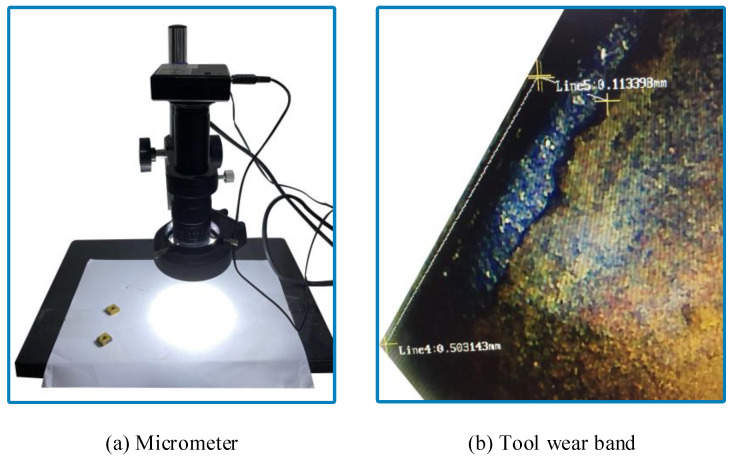
Measurement chart of the tool flank surface wear.

In order to determine the effectiveness of the proposed method, this paper verifies the fault root cause tracking method using specific tool processing experiments. During the turning process, we monitored and collected the real-time condition characteristic signals of the tool, spindle, tool holder, and guide rail. Due to the relatively long service life of the various components of the CNC lathe, to ensure that the verification was completed under safe conditions, the tool with a higher fault and replacement frequency was selected as the fault input point. To accelerate the wear of the tool, we turned off the cutting fluid to accelerate the tool fault during the experimental processing. The specific experimental steps are as follows:

(1) In this test, a new turning tool model YBC251 was selected for the external turning of the workpiece. The specific processing parameters are shown in Table 9, and the cutting processing program code is shown in Figure 21. The running time of each cutting program is 15 s.
(2) After cutting for 15 s, the cutting fluid was turned off, as a result of which the tool showed rapid wear due to high temperature and friction.
(3) The cutting continued for 15 s and promptly ended.
(4) The tool was removed, and a micrometer (Figure 22a) was used to perform measurements. The wear condition of the tool under the micrometer lens is shown in Figure 22b. If the tool did not fail after the measurement, it was necessary to continue turning without cutting fluid until it failed. The measurement results of the tool flank surface wear amount *VB* were recorded, and the corresponding information entropy values were calculated. The results are summarized as shown in Table 10.

During the fault verification experiment, a micrometer was used to measure the wear condition of the tool. After completing a turn in accordance with the cutting program, the tool was removed to measure the amount of wear. In this experiment, the value of the wear amount *VB* was measured four times. Among them, the value of the third measurement is 0.28 mm, which is close to the tool fault standard of 0.3 mm. At this time, the information entropy value calculated by the tool vibration signal is 5.503 bits. When the tool reaches the fault criterion, the calculated information entropy is 5.441 bits. This is basically consistent with the preset fault threshold of 5.489 bits. In the actual application, when the measured information entropy value is close to 5.489 bits, the tool can be replaced. The information entropy reflects the wear amount of the tool; without disassembling the tool to measure the wear amount, the entire state of the tool can be monitored, effectively preventing the effect of the failed tool on the quality of the processed workpiece.

**Figure 23:**
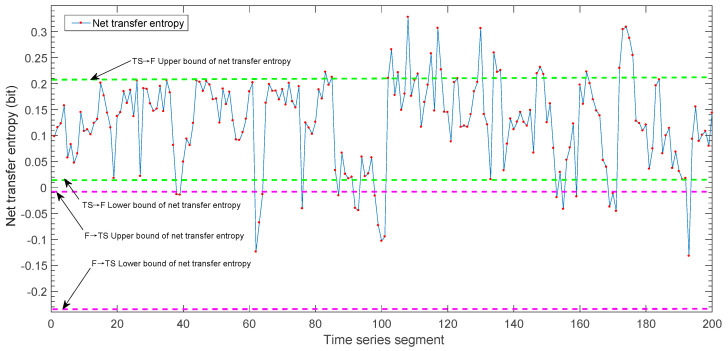
The change-trend graph of the net transfer entropy of the information from the tool holder to the guide rail.

On this basis, to verify the effectiveness of the fault root cause tracking method, the changes in the net transfer entropy of the information on the connecting edges between the mechanical components are monitored. We can obtain the trend chart of the net transfer entropy of the information from the tool holder to the guide rail by taking the information transmission in the two directions of the connecting edge of the guide rail (the tool holder $TE_{F\to TS}$ and the guide rail $TE_{TS\to F}$) as examples. The details are shown in Figure 23.

As shown in Figure 23, in the 100th time series, the value of the information net transfer entropy of the connected edge exceeds the upper limit of the threshold interval. Thus, there is abnormal information flowing from the tool holder to the guide rail. According to the fault tracking process, we continued to calculate the net transfer entropy of the information between the tool and the guide rail. The results also showed that the information net transfer entropy of the connecting edge is 1.4776 in the 100th time series, which exceeds the upper limit of $TE_{T\to TS}$. We conducted a variance analysis for net transfer entropy before and after the 100th time series. The data point before the 100th time series is taken as sequence B1, and the data point after is taken as sequence B2. The normal test results for B1 and B2 are shown in Table 11.

According to the test results, the *p*-value of the significance test is greater than 0.05, which means that the two series are in accordance with the normal distribution.

The purpose of the *t*-test is to infer whether there is a significant difference in the mean between two populations using independent samples from both populations. Here, we calculated the observed values of t and the corresponding *p*-values, as shown in the Table 12 below.

The observed value of the *t*-test statistic is −2.831, and the corresponding bilateral test *p* value is 0.005, which is less than 0.05. This indicates that B_1_ and B_2_ are significantly different, which is consistent with the analysis results in Figure 23, indicating that the net transfer entropy has a sudden change in the 100th time series. Thus, it is determined that there is abnormal information flowing from the tool to the tool holder. According to the fault root cause tracking process, we continue to calculate the net transfer entropy of information between the tool and the spindle near the 100th time series. The calculation result of $TE_{T\to D}$ is 0.5093, which exceeds the upper limit of its threshold interval. Thus, it can be judged that there is abnormal information flowing from the tool into the spindle.

**Figure 24:**
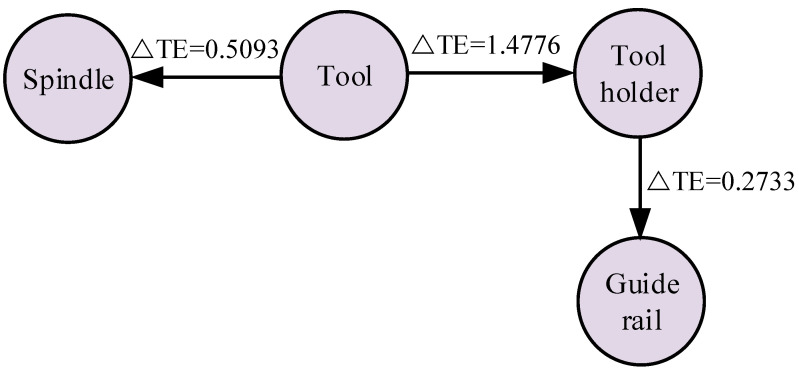
Information transmission model of mechanical components.

As shown in Table 13, the transmission relationship of abnormal information between the components can be obtained according to the process of tracking the root cause of the mechanical fault of the CNC lathe. The information transmission model between mechanical components when the tool fails is shown in Figure 24.

As shown in the flow direction of the information transmission model in Figure 24, the tool holder is not the root cause of the fault, and the starting point of the abnormal information flow is the tool. This means that, according to the process of the mechanical fault root cause tracking method, it can be diagnosed that the tool caused the abnormal information, which is in line with the actual situation. Thus, the effectiveness of the method proposed in this paper is verified.

## 5. Conclusions

This paper proposes a fault root cause tracking method for a CNC lathe’s mechanical components based on information transmission. This research provides a timely and accurate fault diagnosis method for NC lathes during production. Compared with the existing diagnostic techniques, the proposed method uses the collected signals to carry out real-time condition monitoring and fault warning on the lathe, and the results show that the diagnosis is more efficient. The development of this research provides solutions for the troubleshooting and health maintenance of CNC machine tools, thus making fault diagnosis more practical and feasible, while also providing theoretical and technical support for the research on the reliability growth of CNC lathes. The work of this paper can be summarized as follows:

(1) The CNC lathe was divided into subsystems according to its functional structure, mapping relationship, and working principle. Through the analysis of the mechanical component coupling relationship, an undirected graph model of the mechanical component coupling relationship was established to clarify the connection relationship between the mechanical components as well as lay the foundation for the construction of the characteristic signal acquisition platform of the mechanical components.
(2) An experimental platform for signal acquisition of CNC lathes was built, and the corresponding characteristic signals were collected according to the characteristics of the motion state of the mechanical components. The PAA algorithm was used to perform coarse-grained dimensionality reduction processing on the characteristic signal after noise reduction, thus reducing the complexity of signal processing while retaining the original characteristics of the signal.
(3) Based on the operating information of the lathe, the transfer entropy theory was introduced to calculate the net transfer entropy of information between mechanical components, and the net transfer entropies were used to characterize the intensity of information transfer. The positive and negative transfer entropies were used as the basis for judging the direction of the connecting edge, after which the information transmission model of mechanical components was established.
(4) During the operation of the lathe, the net transfer entropy of the information between the mechanical components fluctuated within a certain range. Therefore, this paper used the moving window method to determine the probability threshold interval of the net transfer entropy of information between mechanical components, thereby providing a basis for fault warning and root cause tracking.
(5) By establishing the corresponding relationship between tool wear and information entropy, the critical point of phase transition was determined, and the fault warning of the tool during the machining process was realized. Combined with the information transmission model, we judged whether the inflow and outflow of information exceeded the probability threshold interval of the information net transfer entropy, assessed the flow direction of abnormal information, and traced the root cause of the fault. In addition, the effectiveness of the proposed method was verified by tool processing experiments.

Despite the abovementioned research results obtained in this paper, there are still some limitations to the current work. First, the method established in this paper is only applicable to mechanical parts where relevant information can be collected. For some parts where it is difficult to arrange sensors, we did not include them in the analysis objects. Second, for some possible degenerate failure processes, the early warning sensitivity of this method is low, which may be difficult to identify in time. In view of the lack of current research, this method can be improved in the following aspects in the future:

(1) Consider adding the electrical signal of the equipment system and the temperature change information in the process of processing as monitoring objects, thus establishing the correlation between the mechanical parts and the electrical system.
(2) The influence of the degradation of each mechanical part on the information transfer is often ignored when determining the probability threshold interval of the net information transfer entropy among mechanical parts. In the future, the degradation model of mechanical parts should be combined with the information transfer model; for example, the Archard model should be combined to analyze the tool wear process.
(3) The data processing workload of the proposed method may involve complications when it is used. Thus, in the future, the method can be integrated into software to provide greater convenience when using it and promote its further application in factories.

## Figures and Tables

**Table 1:** Parameter setting of the moving window method.

Spindle Speed	Sampling Frequency	Window Width	Step Length
1000 r/min	5000 HZ	600	480

**Table 2:** Division of the CNC lathe subsystems.

Serial Number	Subsystem Code
1	Auxiliary system-A
2	Foundation components-B
3	Control system-C
4	Transmission system-D
5	Electrical system-E
6	Feed system-F
7	Hydraulic system-H
8	Servo system-S
9	Tool holder system-TS
10	Tool-T

**Table 3:** Dynamic information characteristics of the acquisition components.

Component	Characteristic	Signal Type
D	Rotary movement; radial runout affects workpiece processing.	Displacement signal (radial)
T	The force is greater during the cutting movement, and the impact is obvious.	Vibration signal
TS	Complete the tool feed; the impact is greater; and the structure is complex.	Vibration signal
F	Support the tool holder to complete the translational movement; the impact is greater.	Vibration signal

**Table 4:** Sensor parameters.

Model	Indicator	Range	Resolution	Sampling Frequency
5E101	Displacement	0–1 mm	0.0001 mm	0.05–5000 HZ
1A313	Acceleration	(−120, +120) g	0.0005 g	0.5–5000 HZ

**Table 5:** Comparison table of the signal characteristics of different measuring points.

Measuring Point Position	Signal Frequency Distribution	Root Mean Square	Peak-to-Peak Value
Point 1	490/590/690 HZ	0.048	0.815
Point 2	100/230/340/410/490/580/680 HZ	0.217	2.920
Point 3	220/490/690 HZ	0.144	2.468
Point 4	100/340/490 HZ	0.144	2.012

**Table 6:** Processing parameters for turning the outer circle.

Spindle Speed	Back Cutting Depth	Feed Speed	Tool Model
1000 r/min	2 mm	0.3 mm/r	YBC251

**Table 7:** Net transfer entropy value of information on the connecting edge of each mechanical component.

The Connecting Edge	Net Transfer Entropy (Bit)
$TE_{D\to T}$	0.1931
$TE_{T\to TS}$	0.2577
$TE_{F\to TS}$	0.1876

**Table 8:** Probability threshold interval of information net transfer entropy of each connected edge under two different processing states.

Processing State	The Connecting Edge	Probability Threshold Interval of Information Net Transfer Entropy (Bit)
Simultaneous feed in X and Z directions	$TE_{D\to T}$	[0.1051, 0.4742]
	$TE_{T\to D}$	[0.0137, 0.3815]
	$TE_{T\to TS}$	[0.1352, 1.3011]
	$TE_{TS\to T}$	[0.2010, 1.0352]
	$TE_{F\to TS}$	[0.1109, 0.6233]
	$TE_{TS\to F}$	[0.0921, 0.5772]
Z direction feed	$TE_{D\to T}$	[0.0827, 0.3677]
	$TE_{T\to D}$	[0.0275, 0.2286]
	$TE_{T\to TS}$	[0.1109, 1.1022]
	$TE_{TS\to T}$	[0.1302, 0.9855]
	$TE_{F\to TS}$	[0.0091, 0.2337]
	$TE_{TS\to F}$	[0.0153, 0.2086]

**Table 9:** Experimental processing parameters.

Spindle Speed	Back Cutting Depth	Feed Speed	Tool Model	Workpiece Material
1000 r/min	2 mm	0.5 mm/r	YBC251	45#steel

**Table 10:** Summary of the tool wear degree and information entropy results.

Serial Number	Flank Surface Wear Amount VB (mm)	Information Entropy of the Tool
1	0.03	5.835
2	0.17	5.766
3	0.28	5.503
4	0.33	5.441

**Table 11:** K-S test.

	B_1_	B_2_
Significance test *p*-value	0.148	0.197

**Table 12:** Variance analysis.

	Levene-Test		*t*-Test	
	F	Sig	t	Sig
Equal variances assumed	19.098	0.001	−2.831	0.005
Equal variances not assumed			−2.831	0.005

**Table 13:** Flow direction and status category of abnormal information for each component.

Code	Direction	Status Category
D	Inflow	Affected components
T	Outflow	Root cause of fault
TS	Inflow/Outflow	Affected components/Transmit fault information
F	Inflow	Affected components

## Data Availability

The relevant data of this study involves project confidentiality and is currently unavailable for public access.

## References

[1] Zhang Y.-Z., Liu J.-T., Shen G.-X., Long Z., Sun S.-G. (2017). Reliability Evaluation of Machine Center Components Based on Cascading Failure Analysis. Chin. J. Mech. Eng..

[2] Maurya M.R., Rengaswamy R., Venkatasubramanian V. (2004). Application of signed digraphs-based analysis for fault diagnosis of chemical process flowsheets. Eng. Appl. Artif. Intell..

[3] Smaili R., El Harabi R., Abdelkrim M. (2017). Design of fault monitoring framework for multi-energy systems using Signed Directed Graph. IFAC-PapersOnLine.

[4] Huang X., Gao J., Gao Z. (2012). Precise signed digraph modelling based on causal dependence identification. Proc. Inst. Mech. Eng. Part E J. Process. Mech. Eng..

[5] James A.T., Gandhi O.P., Deshmukh S.G. (2017). Fault diagnosis of automobile systems using fault tree based on digraph modeling. Int. J. Syst. Assur. Eng. Manag..

[6] Pózna A., Fodor A., Gerzson M., Hangos K. (2018). Colored Petri net model of electrical networks for diagnostic purposes. IFAC-PapersOnLine.

[7] Fatma L., Ghabi J., Dhouibi H. (2020). Applying Interval Fuzzy Petri Net to Failure Analysis. Int. J. Serv. Sci. Manag. Eng. Technol..

[8] Peng R. (2018). Reliability of interdependent networks with cascading failures. Eksploat. Niezawodn.-Maint. Reliab..

[9] Duan D., Lv C., Si S., Wang Z., Li D., Gao J., Havlin S., Stanley H.E., Boccaletti S. (2019). Universal behavior of cascading failures in interdependent networks. Proc. Natl. Acad. Sci. USA.

[10] Hosseinalipour S., Mao J., Eun D.Y., Dai H. (2019). Prevention and Mitigation of Catastrophic Failures in Demand-Supply Interdependent Networks. IEEE Trans. Netw. Sci. Eng..

[11] Zhou Q., Yan P., Liu H., Xin Y., Chen Y. (2017). Research on a configurable method for fault diagnosis knowledge of machine tools and its application. Int. J. Adv. Manuf. Technol..

[12] Feng J., Yao Y., Lu S., Liu Y. (2020). Domain Knowledge-Based Deep-Broad Learning Framework for Fault Diagnosis. IEEE Trans. Ind. Electron..

[13] Schmid M., Kneidinger H.-G., Endisch C. (2020). Data-Driven Fault Diagnosis in Battery Systems through Cross-Cell Monitoring. IEEE Sens. J..

[14] Jung D., Ng K.Y., Frisk E., Krysander M. (2018). Combining model-based diagnosis and data-driven anomaly classifiers for fault isolation. Control. Eng. Pract..

[15] Slimani A., Ribot P., Chanthery E., Rachedi N. (2018). Fusion of Model-Based and Data-Based Fault Diagnosis Approaches. IFAC-PapersOnLine.

[16] Gangsar P., Tiwari R. (2020). Signal based condition monitoring techniques for fault detection and diagnosis of induction motors: A state-of-the-art review. Mech. Syst. Signal Process..

[17] Khan R., Khan S.U. (2018). Design and implementation of an automated network monitoring and reporting back system. J. Ind. Inf. Integr..

[18] Zhou Q., Yan P., Liu H., Xin Y. (2017). A hybrid fault diagnosis method for mechanical components based on ontology and signal analysis. J. Intell. Manuf..

[19] Alam Shifat T., Hur J.-W. (2021). ANN Assisted Multi Sensor Information Fusion for BLDC Motor Fault Diagnosis. IEEE Access.

[20] Hoang D.T., Tran X.T., Van M., Kang H.J. (2021). A Deep Neural Network-Based Feature Fusion for Bearing Fault Diagnosis. Sensors.

[21] Chen X., Zhang B., Gao D. (2020). Bearing fault diagnosis base on multi-scale CNN and LSTM model. J. Intell. Manuf..

[22] Gunerkar R.S., Jalan A.K., Belgamwar S.U. (2019). Fault diagnosis of rolling element bearing based on artificial neural network. J. Mech. Sci. Technol..

[23] Hou Q., Wang L., Lu N.Y., Jiang B., Lu J.H. A FDD method by combining transfer entropy and signed digraph and its application to air separation unit. Proceedings of the 2010 11th International Conference on Control Automation Robotics & Vision.

[24] Al-Jonid K., Jiayang W., Mohammed N., Nurudeen M. A new fault classification model for prognosis and diagnosis in CNC machine. Proceedings of the 2013 25th Chinese Control and Decision Conference (CCDC) 2013.

[25] Colasante A., Ceccacci S., Talipu A., Mengoni M. (2019). A Fuzzy Knowledge-Based System for Diagnosing Unpredictable Failures in CNC Machine Tools. Procedia Manuf..

[26] Serin G., Sener B., Ozbayoglu A.M., Unver H.O. (2020). Review of tool condition monitoring in machining and opportunities for deep learning. Int. J. Adv. Manuf. Technol..

[27] Lindner B., Auret L., Bauer M., Groenewald J.W. (2019). The Comparative analysis of Granger causality and transfer entropy to present a decision flow for the application of oscillation diagnosis. J. Process. Control..

[28] Li G., Qin S.J., Yuan T. (2016). Data-driven root cause diagnosis of faults in process industries. Chemom. Intell. Lab. Syst..

[29] He R., Chen G., Sun S., Dong C., Jiang S. (2020). Attention-Based Long Short-Term Memory Method for Alarm Root-Cause Diagnosis in Chemical Processes. Ind. Eng. Chem. Res..

[30] Xie P., Yang F.M., Li X.X., Yang Y., Chen X.L., Zhang L.T. (2016). Functional coupling analyses of electroencephalogram and electromyogram based on variational mode decomposition-transfer entropy. Acta Phys. Sin..

[31] Zou S., Qiu T., Huang P., Bai X., Liu C. (2020). Constructing Multi-Scale Entropy Based on the Empirical Mode Decomposition(EMD) and Its Application in Recognizing Driving Fatigue. J. Neurosci. Methods.

[32] Zhang Z., Wang Y., Wang K. (2012). Fault diagnosis and prognosis using wavelet packet decomposition, Fourier transform and artificial neural network. J. Intell. Manuf..

[33] Tangjitsitcharoen S., Saksri T., Ratanakuakangwan S. (2013). Advance in chatter detection in ball end milling process by utilizing wavelet transform. J. Intell. Manuf..

[34] Liu S., Hu Y., Li C., Lu H., Zhang H.-C. (2015). Machinery condition prediction based on wavelet and support vector machine. J. Intell. Manuf..

[35] Kim J., Lee H., Jeon J.W., Kim J.M., Lee H.U., Kim S. (2020). Stacked Auto-Encoder Based CNC Tool Diagnosis Using Discrete Wavelet Transform Feature Extraction. Processes.

[36] Ribeiro M., Henriques T., Castro L., Souto A., Antunes L., Costa-Santos C., Teixeira A. (2021). The Entropy Universe. Entropy.

[37] Theodorou E.A. (2015). Nonlinear Stochastic Control and Information Theoretic Dualities: Connections, Interdependencies and Thermodynamic Interpretations. Entropy.

[38] Li J., Pan Q. (2020). A New Belief Entropy in Dempster–Shafer Theory Based on Basic Probability Assignment and the Frame of Discernment. Entropy.

[39] Choudhury R.A., McRoberts N. (2020). Characterization of Pathogen Airborne Inoculum Density by Information Theoretic Analysis of Spore Trap Time Series Data. Entropy.

[40] Schreiber T. (2000). Measuring Information Transfer. Phys. Rev. Lett..

[41] Qian J., Guo X., Deng Y. (2016). A novel method for combining conflicting evidences based on information entropy. Appl. Intell..

[42] Britton T., Pardoux E. (2019). Chapter 2 Markov Models. Stochastic Epidemic Models with Inference.

[43] Xin G., Hamzaoui N., Antoni J. (2018). Semi-automated diagnosis of bearing faults based on a hidden Markov model of the vibration signals. Measurement.

[44] Nichols J., Seaver M., Trickey S. (2006). A method for detecting damage-induced nonlinearities in structures using information theory. J. Sound Vib..

[45] Yao W., Wang J. (2017). Multi-scale symbolic transfer entropy analysis of EEG. Phys. A Stat. Mech. Appl..

[46] He J., Shang P. (2017). Comparison of transfer entropy methods for financial time series. Phys. A Stat. Mech. Appl..

[47] Liu J., Wang J., Liu X., Ma T., Tang Z. (2021). MWRSPCA: Online fault monitoring based on moving window recursive sparse principal component analysis. J. Intell. Manuf..

[48] Sun Y., Qin W., Zhuang Z., Xu H. (2021). An adaptive fault detection and root-cause analysis scheme for complex industrial processes using moving window KPCA and information geometric causal inference. J. Intell. Manuf..

[49] Wang R., Gao X., Gao J., Gao Z., Kang J. (2018). An information transfer based novel framework for fault root cause tracing of complex electromechanical systems in the processing industry. Mech. Syst. Signal Process..

[50] Bauer M., Cox J.W., Caveness M.H., Downs J.J., Thornhill N.F. (2006). Finding the Direction of Disturbance Propagation in a Chemical Process Using Transfer Entropy. IEEE Trans. Control. Syst. Technol..

[51] (2004). Metal Cutting Principles, Second Edition, American Society of Mechanical Engineers, New York. https://www.proquest.com/magazines/metal-cutting-principles-second-edition/docview/230184116/se-2?accountid=11718.

[52] Zhu R., Dong H. (2019). Fundamentals of Mechanical Manufacturing Technology.

[53] Qiu Y., Wang B. (2019). Practical Technical Manual for Metal Cutting Tools.

